# Alzheimer’s disease neuropathological change three decades after iatrogenic amyloid-β transmission

**DOI:** 10.1007/s00401-021-02326-y

**Published:** 2021-05-28

**Authors:** Zane Jaunmuktane, Gargi Banerjee, Simon Paine, Adrian Parry-Jones, Peter Rudge, Joan Grieve, Ahmed K. Toma, Simon F. Farmer, Simon Mead, Henry Houlden, David J. Werring, Sebastian Brandner

**Affiliations:** 1 Division of Neuropathology, National Hospital for Neurology and Neurosurgery, University College London NHS Foundation Trust, London, WC1N 3BG UK; 2 Department of Clinical and Movement Neurosciences and Queen Square Brain Bank for Neurological Disorders, Queen Square Institute of Neurology, University College London, London, WC1N 3BG UK; 3 MRC Prion Unit at UCL, Institute of Prion Diseases, University College London, London, W1W 7FF UK; 4 Neuropathology Laboratory, Queen’s Medical Centre, Nottingham University Hospitals NHS Trust, Nottingham, NG7 2UH UK; 5 Geoffrey Jefferson Brain Research Centre, Manchester Academic Health Science Centre, Northern Care Alliance, University of Manchester, Manchester, M13 9PL UK; 6 Division of Cardiovascular Sciences, School of Medicine, Faculty of Biology, Medicine and Health, The University of Manchester, Manchester, M13 9PL UK; 7 National Prion Clinic, National Hospital for Neurology and Neurosurgery, University College London Hospitals NHS Foundation Trust, London, WC1N 3BG UK; 8 Victor Horsley Department of Neurosurgery, National Hospital for Neurology and Neurosurgery, University College London NHS Foundation Trust, London, WC1N 3BG UK; 9 Department of Neurology, National Hospital for Neurology and Neurosurgery, University College London NHS Foundation Trust, London, WC1N 3BG UK; 10 Neurogenetics Laboratory, National Hospital for Neurology and Neurosurgery, University College London NHS Foundation Trust, London, WC1N 3BG UK; 11 Department of Neuromuscular Diseases, UCL Queen Square Institute of Neurology, London, WC1N 3BG UK; 12 Stroke Research Centre, Department of Brain Repair and Rehabilitation, Queen Square Institute of Neurology, University College London, London, WC1N 3BG UK; 13 Department of Neurodegenerative Disease, Queen Square Institute of Neurology, University College London, London, WC1N 3BG UK

Human (iatrogenic) transmission of amyloid-β (Aβ) pathology has been shown in brain biopsy or autopsy tissues in patients with and without iatrogenic Creutzfeldt–Jakob disease (iCJD) [1, 5–18, 20–22] and these Aβ seeds have been detected in the archival vials containing human cadaver-derived growth hormone (hcGH) [7, 19]. Whilst tau seeds were also found in these hcGH vials [7, 19], to date, no substantial tau pathology has been observed in patients with iCJD, iatrogenically transmitted Aβ pathology or both.

Here we show that a significant tau pathology, similar to that seen in patients with Alzheimer’s disease, can develop in patients with iatrogenic Aβ pathology after incubation periods exceeding 3 decades.

**Fig. 1:**
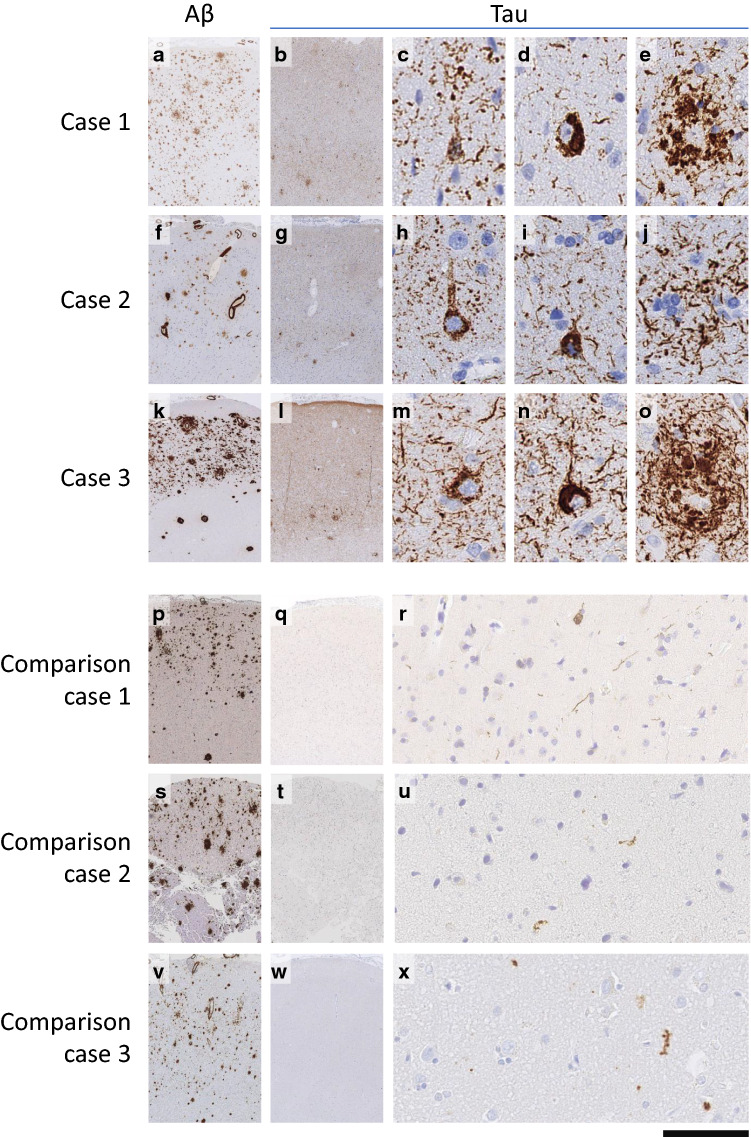
Amyloid-β and tau pathology in cases 1–3 with an overview of Aβ pathology (**a**, **f**, **k**), tau pathology (**b**, **g**, **l**) and high power details of pre-tangles (**c**, **h**, **m**), tangles (**d**, **i**, **n**) and neuritic plaques (**e**, **j**, **o**), detected with antibodies against Amyloid-β (Clone 6F3D, DAKO M0872) and Tau (Clone AT8, Thermo MN1020). In three comparison cases with comparable incubation periods and similarly widespread Aβ load (**p**, **s**, **v**), there is minimal tau pathology: comparison case 1, incubation 37 years (case 1 in [1]; **q**, **r**, comparison case 2, incubation 35 years (case 3 in [16]) **t**, **u**, and comparison case 3, incubation 36 years (case 4 in [16]) **w**, **x**. Comparison cases are also highlighted with an asterisk (*) in Fig. 2

Case 1: a 46-year-old male presented with a 12-month history of cognitive decline, progressive ataxia and myoclonus. He had a medulloblastoma resected at the age of 4 years, but it is not known if a dura patch was used. The patient had learning difficulties since the radio-chemotherapy of his tumour but several months after a caudate nucleus haemorrhage at age 44, he developed gradual cognitive decline. A right frontal brain biopsy showed leptomeningeal and cortical Aβ angiopathy (CAA), parenchymal amyloid-β with diffuse deposits and plaques with central amyloid cores (Fig. 1a), and a tauopathy forming a meshwork of neuropil threads, pre-tangles, tangles and neuritic plaques (Fig. 1b–e). The patient died at the age of 47. *APOE* testing was not performed, but genetic testing did not identify any pathogenic mutations in 17 genes associated with neurodegenerative diseases [2], notably including the amyloid precursor protein gene *(APP)* (including duplication of *APP*), Presenilin 1 *(PSEN1)*, Presenilin 2 *(PSEN2)* and microtubule-associated protein tau *(MAPT)*.

Case 2: a 39-year-old male presented with intracerebral haematoma and underwent emergency blood-clot evacuation. As a child, he had multiple haemangiomas, embolised at the age of 3 years (retro-auricular, embolisation agent unknown), 4 years [facial, embolised with lyophilised cadaveric dura (hcDM)], 8 years (re-embolisation of the facial lesion, with Ivalon (polyvinyl alcohol particles) and at the age of 9 (re-embolisation of the retro-auricular lesion with Ivalon). A parietal lobe biopsy from the perihaematoma region (Fig. 1f–j) showed leptomeningeal and cortical Aβ angiopathy, parenchymal Aβ with diffuse deposits and plaques with central amyloid cores, and tau pathology with a loose meshwork of neuropil threads, occasional pre-tangles and rare tangles and neuritic plaques. At follow-up, the patient had no cognitive impairment. *APOE* genotype was ε2/ε3, and no genetic risks or pathogenic mutations associated with early Aβ pathology were identified [2].

Case 3: a 45-year-old female presented with a convexity subarachnoid haemorrhage. As a child, she underwent multiple embolisations of facial haemangiomas including lyophilised cadaveric dura at age of 6 years. A right frontal brain biopsy (Fig. 1k–o) showed leptomeningeal and cortical Aβ angiopathy, parenchymal Aβ with diffuse deposits and plaques with central amyloid cores, and a widespread cortical tauopathy with neuropil treads, pre-tangles, tangles, and neuritic plaques. At the time of the biopsy, the patient had no cognitive impairment. Genetic testing showed an *APOE* ε3/ε3 genotype, and a *NOTCH3* c.2183G>A (p.(Arg728His)) variant of unknown significance but no genetic risks associated with early Aβ pathology [2].

**Fig. 2:**
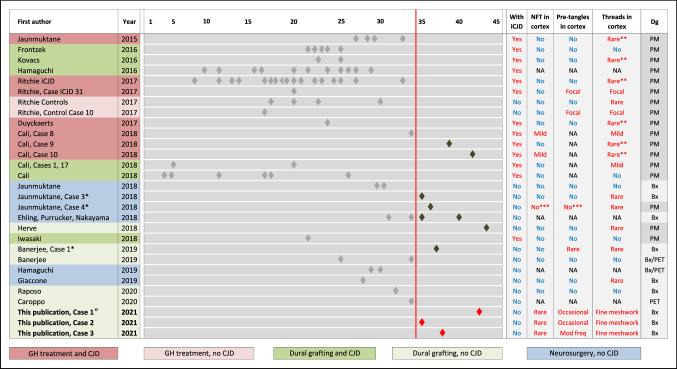
Visualisation of iatrogenic Aβ pathology incubation times in the current and in published studies. Left columns, first author and publication year, and case ID in the respective publication, where indicated. Centre, timeline of reported incubation times. Each diamond indicates a published case. Cases with incubation times of 35 and more years are highlighted in dark grey (published) and red (this study). The reported presence of tau pathology is indicated in the four columns on the right. The column on the far right indicates the sample type (Bx—diagnosed on biopsy; PM—diagnosed on post-mortem material; PET—diagnosed on in vivo PET imaging). * (leftmost column) indicate three comparison cases shown in Fig. 1; ** (column “threads in cortex”) highlight cases, where rare neocortical threads or granular tau pathology were reported in the context of abnormal prion protein pathology; *** (columns “NFT (neurofibrillary tangles) in cortex” and “pre-tangles in cortex”) corresponds to a case in which tau pathology is seen in the medial temporal lobe but not in the neocortex. °For case 1 it is unknown if a cadaver-derived dural graft was used during neurosurgery

All three cases reported here have in common particularly long incubation times, exceeding 35 years for iatrogenically transmitted Aβ. No patient had a history of brain trauma, and neocortical tau pathology is extremely rare in young adults [3]. The few cases with long incubation periods reported to date (Fig. 2) do not show an obvious correlation between the extent of parenchymal Aβ pathology or the type of Aβ plaques (diffuse or plaques with central cores). Notably, plaques with central cores but without associated tau positive neurites are not uncommon in patients with iatrogenically transmitted Aβ [15].

Whilst this study cannot answer if the tauopathy was transmitted or is a consequence of Aβ pathology, the observations described here are important as they show that tau pathology can develop in patients with iatrogenically transmitted Aβ.

Our observations give some insight into the temporal development of tau pathology. Given that substantial tau pathology in non-iCJD patients has not been seen previously (Fig. 2), at least 35 years appears to be necessary for the development of neocortical neurofibrillary tangle and widespread thread tau pathology. However, tau pathology of similar severity is not present in all patients with iatrogenic Aβ pathology with an incubation period exceeding 35 years. The three cases reported by us previously [1, 16], with equally long incubation, and similarly widespread parenchymal Aβ pathology did not show such severe tau pathology in the neocortical biopsies, although one of these cases for which whole brain tissue was available for analysis, did show tau pathology in the medial temporal lobe corresponding to Braak & Braak stage II [16].

This study highlights the importance of enquiring about previous potential iatrogenic exposure and searching medical records for treatments with hcGH or interventions using hcDM in patients with early-onset intracranial (intracerebral or non-aneurysmal subarachnoid) haemorrhages as hcDM was used not only for neurosurgical repairs but also for interventional embolisation and other surgeries [4]. The severe, often fatal, haemorrhagic consequences of iatrogenic vascular Aβ pathology have been documented [1, 6, 8, 10, 11, 13, 14, 16, 18, 21] (Fig. 2). The cases reported here indicate that in addition to CAA and parenchymal Aβ pathology, tau pathology, indistinguishable from Alzheimer’s type changes, can develop after particularly long incubation periods.
